# PP1 Forms an Active Complex with TLRR (lrrc67), a Putative PP1 Regulatory Subunit, during the Early Stages of Spermiogenesis in Mice

**DOI:** 10.1371/journal.pone.0021767

**Published:** 2011-06-30

**Authors:** Rong Wang, Ann O. Sperry

**Affiliations:** Department of Anatomy and Cell Biology, Brody School of Medicine at East Carolina University, Greenville, North Carolina, United States of America; National Institutes of Health, United States of America

## Abstract

Mammalian spermatogenesis is a highly regulated developmental pathway that demands dramatic rearrangement of the cytoskeleton of the male germ cell. We have described previously a leucine rich repeat protein, TLRR (also known as lrrc67), which is associated with the spermatid cytoskeleton in mouse testis and is a binding partner of protein phosphatase-1 (PP1), an extremely well conserved signaling molecule. The activity of PP1 is modulated by numerous specific regulators of which TLRR is a candidate. In this study we measured the phosphatase activity of the TLRR-PP1 complex in the adult and the developing mouse testis, which contains varying populations of developing germ cell types, in order to determine whether TLRR acts as an activator or an inhibitor of PP1 and whether the phosphatase activity of this complex is developmentally regulated during spermatogenesis. Additionally, we assayed the ability of bacterially expressed TLRR to affect the enzymatic activity of PP1. Furthermore, we examined phosphorylation of TLRR, and elements of the spermatid cytoskeleton during the first wave of spermatogenesis in the developing testis. We demonstrate here that the TLRR complex is associated with a phosphatase activity in adult mouse testis. The relative phosphatase activity of this complex appears to reach a peak at about 21 days after birth, when pachytene spermatocytes and round spermatids are abundant in the seminiferous epithelium of the mouse testis. TLRR, in addition to tubulin and kinesin-1B, is phosphorylated during the first wave of spermatogenesis. These findings indicate that the TLRR-PP1 complex is active prior to translocation of TLRR toward the sperm flagella and that TLRR, and constituents of the spermatid cytoskeleton, may be subject to regulation by reversible phosphorylation during spermatogenesis in murine testis.

## Introduction

The acquisition of cellular polarity is essential for proper differentiation and function of specialized cells and tissues including neurons, epithelial cells, and spermatozoa. Male germ cells undergo a highly orchestrated series of events to transform the unpolarized precursor cell, the spermatogonium, into a highly polarized spermatozoon, in which most of its original cytoplasm and organelles are discarded and only those structures necessary for successful delivery of the nuclear contents to the egg at fertilization are retained. Most of these shape changes occur after meiosis during a phase of spermatogenesis termed spermiogenesis. A hallmark of spermiogenesis is formation of the microtubule manchette that encircles the nucleus and is present while the nucleus condenses and elongates but is disassembled before sperm release into the lumen [1]. The purpose of the manchette is not well defined but has been proposed to aid in the condensation of the spermatid nucleus, in transport of cytoplasm to the distal aspect of the spermatid and to act as a platform for signaling molecules necessary for regulation of spermatid transformation [2]–[4].

We have identified previously a leucine rich repeat protein, highly expressed in the testis, which is associated with the elongating spermatid manchette and with the centrosome [5], [6]. We also determined that this previously uncharacterized protein (TLRR, testis leucine rich repeat; also known as lrrc67) contains a docking site for PP1 and resides in a complex containing this protein in the testis [6]. Furthermore, TLRR interacts with PP1γ2, a testis-specific isoform of PP1 whose activity is required for spermatogenesis [7]. The aim of this study was to determine whether TLRR acts as a regulatory subunit of PP1 in testis to either increase or decrease its activity. In this study, we show that the TLRR complex isolated from the adult testis contains phosphatase activity and that this activity peaks in the developing testis at a time when round spermatids appear in the epithelium and when the interaction between TLRR and PP1 is at its highest level. Although the TLRR-PP1 complex in the testis has phosphatase activity, bacterially expressed TLRR had no affect on PP1 activity *in vitro* suggesting that this protein alone does not regulate PP1 or that post-translational modification of these proteins is required for modulation. We also demonstrate here that TLRR is phosphorylated during the first wave of spermatogenesis in the developing testis along with tubulin and kinesin-1B, both of which we have shown previously to be associated with TLRR in the testis [6].

## Results

### Phosphatase activity is associated with the TLRR complex

**Figure 1:**
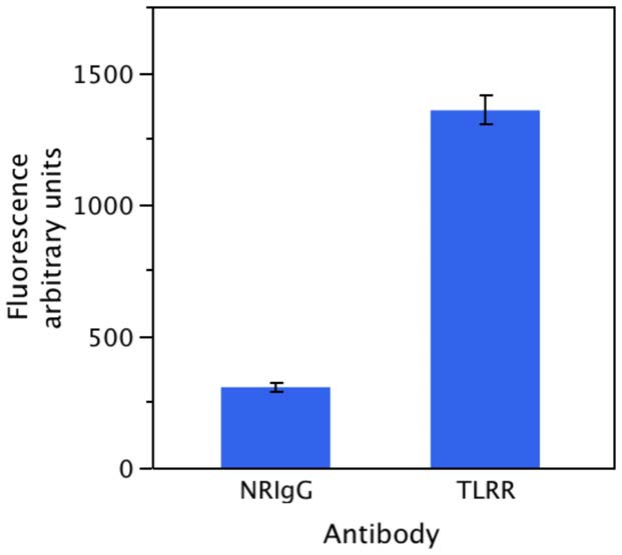
Phosphatase activity is associated with the TLRR complex immunoprecipitated from adult testis lysate. TLRR complex phosphatase activity was measured using the EnzChek® Phophatase Assay Kit (Invitrogen) as described in Materials and Methods. Either TLRR antibody or Normal Rabbit IgG (NRIgG) as negative control were linked to Sepharose beads and incubated with 1 mg (25 µl of 40 µg/µl) CD-1 adult mouse testis lysate. Proteins bound to beads were eluted and added to fluorescence substrate and resultant fluorescence was measured after 60 min. The results are significant at p = 0.01.

We have demonstrated previously that TLRR interacts with PP1 in testis lysate [6]. We predict therefore that the TLRR complex in testis might contain protein phosphatase activity. Testis lysate prepared from adult testis was immunonoprecipitated either with affinity purified anti-TLRR antibody or normal rabbit IgG. Figure 1 illustrates that the TLRR complex isolated by immunoprecipitation from testis lysate contains significant protein phosphatase activity compared to proteins immunoprecipitated with control antibody.

### TLRR-associated phosphatase activity peaks when round spermatids appear in the developing testis

**Figure 2:**
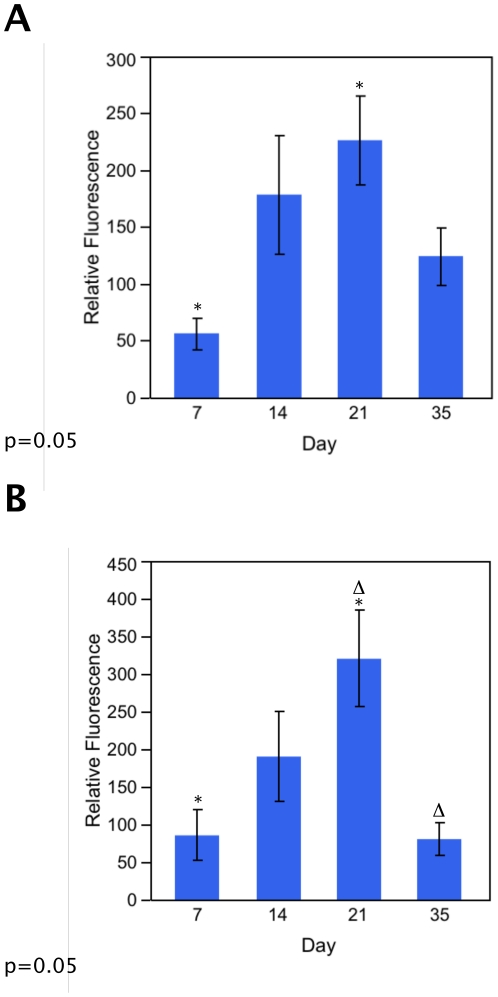
TLRR-associated phosphatase activity varies with developmental stage of the testis. (A) Testis lysate from day 7, 14, 21, 35, or 56 mice was incubated with Sepharose beads linked to TLRR antibody. Protein phosphatase activity of bound proteins was measured as described in Figure 1 using the Enzchek® assay for total phosphatase activity and the results are displayed relative to the activity at 56 days (set as 100%). Protein phosphatase activity at day 7 and day 21 are significantly different at p = 0.05. (B) Testis lysate from day 7, 14, 21, 35, or 56 mice was immunoprecipitated with TLRR antibody beads and the PP1-specific phosphatase activity in bound proteins was measured with Rediplate 96 EnzChek® Serine/Threonine Phosphatase Assay kit (Invitrogen). Fluorescence was measured at 460 nm. PP1 activity is significantly different between day 7 and day 21 (asterisks) and between day 21 and day 35 (deltas). Differences are significant at p = 0.05.

TLRR is located near the nucleus of developing spermatids [5], [6]. In order to determine whether the relative amount of phosphatase activity associated with the TLRR complex varies during the spermatogenic cycle in developing testes, tissue lysates were prepared from testes at different ages after birth: 7 days, 14 days, 21 days, 35 days and 56 days. These time points were chosen because different germ cell types appear approximately at each time: spermatogonia at 7 days, spermatocytes at about 14 days, round spermatids at 21 days, elongating spermatids at 35 days, while all cell types are found in the epithelium at 56 days. The TLRR complex was immunoprecipitated with the TLRR antibody and the total protein phosphatase activity or the PP1 specific activity was measured and is displayed as a percentage of the phosphatase activity at 56 days (Figure 2).

The total protein phosphatase activity associated with the TLRR complex reached its highest point at about 21 days after birth with a statistically different value between 7 days and 21 days (asterisks, Figure 2A). The same trend was seen when PP1 specific activity was assayed in these samples (Figure 2B) with a statistically significant difference observed between the TLRR-associated PP1 activity at 7 days compared to 21 days and between 21 days and 35 days. Lysate isolated from all time points displayed a higher relative phosphatase activity compared to adult mouse testis lysate as indicated by their positive values. These results indicate that TLRR-associated PP1 activity is highest at the time when round spermatids appear in the seminiferous epithelium.

### TLRR interaction with PP1 is developmentally regulated during the first wave of spermatogenesis

**Figure 3:**
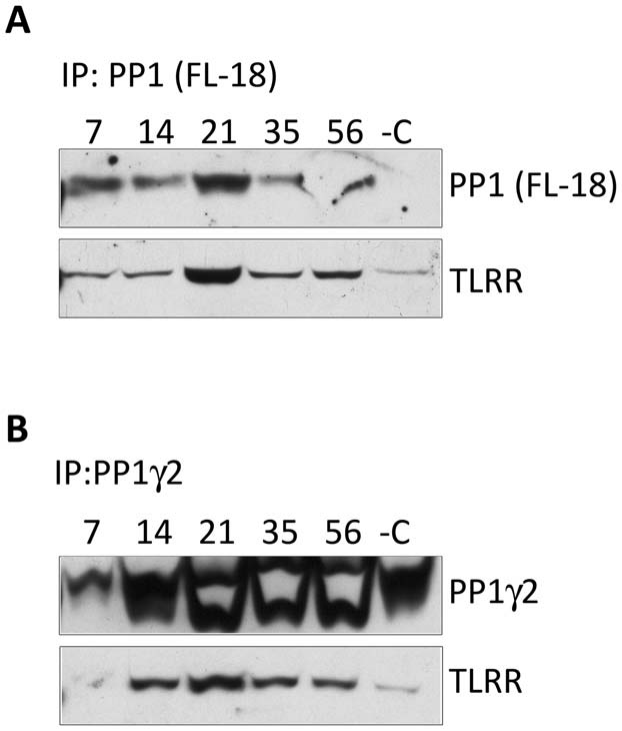
The quantity of TLRR-PP1 complex varies with developmental age of the testis. 2 mg testis lysate from animals at the indicated days after birth was immunoprecipitated with either the pan-PP1 antibody FL-18 (A) or an antibody specific to the gamma 2 isoform (B). Bound proteins were separated by SDS-PAGE, transferred to membrane and probed with the corresponding antibody or an antibody to TLRR. In the negative control (-C) normal rabbit IgG was used in place of the PP1 antibody.

We have demonstrated previously that TLRR interacts with PP1, including the gamma 2 isoform of this phosphatase [6]. We next wanted to determine whether this association is developmentally regulated during initiation of spermatogenesis in the testis and whether interaction between these proteins correlates with the appearance of individual cell types within the testis. PP1 was immunoprecipitated from testis lysate obtained from 7, 21, 35, or 56-day-old mice using either the pan-PP1 antibody FL-18 (Figure 3A) or a gamma 2 specific antibody (Figure 3B) and blots incubated with TLRR antibody. The interaction between TLRR and PP1 reaches its highest point at about 21 days after birth (Figure 3A), consistent with the increased phosphatase activity observed at this time. Correspondingly, TLRR association with PP1γ2 reaches a peak at about the same developmental time point (Figure 3B). These results are consistent with the proposal that TLRR interacts with PP1 in a developmentally regulated manner in the testis.

### TLRR expression during mouse testis development is complementary to that of PP1γ2

**Figure 4:**
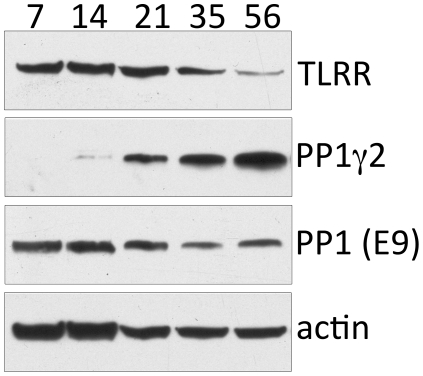
The expression profiles of TLRR and PP1γ2 are conversely related during development of the testis. 50 µg testis lysate from animals at the indicated days after birth was separated by SDS-PAGE, transferred to membrane, and probed with antibodies specific for the indicated proteins.

Our co-immunoprecipitation experiments demonstrate that the TLRR-PP1 complex is enriched in the testis at 21 days after birth when spermatocytes and round spermatids are most abundant. In order to determine whether the observed increase at 21 days reflects a developmental regulation of TLRR expression during initiation of spermatogenesis in the testis, we measured the amount of TLRR, total PP1, and PP1γ2 at the same time points after birth (Figure 4). TLRR decreases as a percentage of total protein in the developing testis while the opposite is true of PP1γ2 expression, which reaches a peak in adult testis, as has been reported by other investigators [8], [9]. Total PP1, as detected by the pan-PP1 antibody E9, does not change dramatically during development. Therefore, TLRR-associated phosphatase activity during development coincides with the time period in the seminiferous epithelium when TLRR and PP1γ2 are co-expressed.

**Figure 5:**
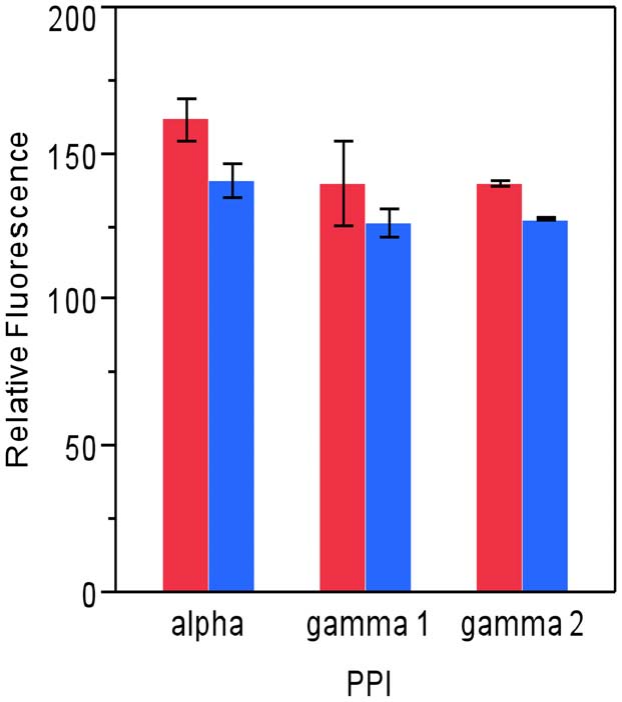
Recombinant TLRR does not affect the phosphatase activity of PP1 isoforms. The activity of His6 tagged PP1 isoforms alone (red bars) or with an equal amount of TLRR (blue bars) was assayed for PP1-specific activity as described in the text using the RediPlate 96 EnzChek® Serine/Threonine Phosphatase Assay kit from Invitrogen.

TLRR is associated with PP1γ2 in testis lysate and this complex reaches its highest point when spermatocytes and round spermatids are abundant in the testis. We expressed TLRR and its putative binding partners PP1α, PP1γ1, and PP1γ2, in bacteria to determine whether recombinant TLRR was able to affect the phosphatase activity of PP1 and whether this effect might be isoform specific using modification of an approach used by others [10]. However, we were unable to detect any significant affect of bacterially expressed TLRR on the phosphatase activity of any PP1 isoform (Figure 5).

### TLRR is phosphorylated in the developing testis along with cytoskeletal subunits and the molecular motor kinesin1-B

**Figure 6:**
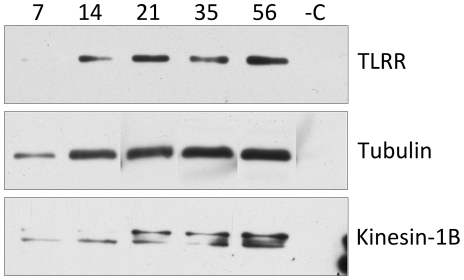
TLRR, kinesin-1B and tubulin are phosphorylated during the first round of spermatogenesis in the developing testis. 2 mg testis lysate from animals at the indicated days after birth was immunoprecipitated with a mixture of anti-phosphoserine antibodies as described in Materials and Methods. Immunoprecipitated proteins were separated by SDS-PAGE, transferred to membrane and blotted with antibodies specific to the proteins indicated to the right. –C indicates proteins immunoprecipitated with normal rabbit IgG.

Our finding that TLRR does not significantly affect PP1 activity could be due to the lack of posttranslational modification of bacterially expressed proteins. Other investigators have suggested that inhibition/activation of PP1 by its modulators is regulated by phosphorylation [11], [12] and PP1 itself is phosphorylated in spermatozoa [13]. We have demonstrated previously that TLRR exists in a large complex containing cytoskeletal polymers and motor proteins and is phosphorylated [6]. In order to determine whether the phosphorylation status of TLRR and associated cytoskeletal proteins changes during spermatogenesis, we immunoprecipitated phosphorylated proteins from testis lysate obtained from animals at different times after birth and blotted for TLRR, kinesin-1B (the kinesin isoform that we have previously shown to be associated with TLRR [6]) and tubulin. TLRR is increasingly phosphorylated during the first round of spermatogenesis in the testis, with a marginal increase in phosphorylation at 21 days after birth (Figure 6). Tubulin phosphorylation increases throughout the first round of spermatogenesis in the testis. Kinesin-1B also demonstrated an increase in phosphorylation during this time course and, interestingly, appears progressively more heterogeneous in size suggesting multiple phosphorylated isoforms as development proceeds in the testis (Figure 6).

## Discussion

TLRR contains a consensus-binding site for PP1 and we have shown by co-immunoprecipitation that it resides in a protein complex in the testis that also contains PP1 isoforms [5], [6]. The studies described here demonstrate that the TLRR complex in the adult testis contains PP1-specific phosphatase activity and that this activity reaches its greatest level when round spermatids appear in the testis, around 21 days after birth. Importantly, the complex containing TLRR and PP1 is also most abundant at this time point. Our observations that the TLRR-PP1 complex is at its highest level when round spermatids appear in the testis and associated PP1 activity also reaches a peak at this time point suggests an increased association and activity in this cell type. This is in contrast to our previous data that TLRR is localized on the manchette and centrosome of elongating spermatids and our proposal that TLRR targets PP1 to these structures in order to regulate associated proteins in the later stages of spermiogenesis. Based on these results, we speculate that association between TLRR and PP1 is required for an event in spermatocytes or round spermatids before the appearance of TLRR on the manchette and its transit to the centrosome in elongated spermatids. PP1 isoforms are differentially expressed in male germ cells with PP1γ2 abundant in the cytoplasm of secondary spermatocytes through mature sperm but also expressed at a lower level in spermatogonia and pachytene spermatocytes [8]. The other isoforms, PP1α and PP1γ1, are also expressed in male germ cells including spermatogonia and spermatocytes, but to a lesser extent in spermiogenesis compared to PP1γ2 [8]. Therefore, TLRR may participate in regulation of PP1 function by interaction with different PP1 isoforms along with cell-type specific factors at different times during spermatogenesis.

The microtubule organization of round spermatids is quite different from that of somatic cells in that a definitive microtubule-organizing center is not obvious [14], [15]. Other investigators have described significant rearrangement of microtubules associated with the spermatid nucleus at about step 7 and have suggested that this reorganization is a precursor to the manchette [15]. TLRR may interact with a specific PP1 isoform in round spermatids in order to regulate downstream targets in preparation for the microtubule rearrangements that follow in later stage spermatids. We have shown previously that the TLRR complex in the testis contains cytoskeletal polymers, molecular motor proteins, and molecular chaperones, that may be potential targets of regulation by the TLRR-PP1 complex [6].

The expression patterns of TLRR and PP1γ2 are strikingly different, with the latter increasing throughout the first wave of spermatogenesis while TLRR expression decreases during this period. The pattern of PP1γ2 expression reflects the fact that this protein is a constituent of the sperm flagella and that spermatozoa represent about 40% of the total cell population in the adult testis [16]. Although TLRR is a homolog of Ppp1r7 (sds22), a known regulator of PP1γ2 in the sperm flagella, its divergent expression suggests that TLRR is not a strict functional homolog of sds22 and may regulate PP1 at an earlier time in spermatocytes and/or round spermatids. PP1γ2 null mice show defects in meiosis and PP1γ2 is found in the nuclei of spermatocytes and round spermatids supporting a role for PP1γ2 in regulating chromosome dynamics during meiosis and/or the initial stages of spermiogenesis [17], [18]. Significantly, the developmental time point enriched in spermatocytes and spermatids contained the highest level of TLRR-associated phosphatase activity and TLRR-PP1 interaction, suggesting a role for this complex in meiosis and/or preparation for chromosomal condensation. Further *in vivo* studies are needed to determine whether TLRR functions with individual PP1 isoforms to regulate activity in different spermatogenic cell types.

Another important finding of this work is that tubulin and kinesin-1B are phosphorylated along with TLRR in developing testis. Both of these proteins are part of the TLRR complex in testis [6]. Our finding that tubulin is phosphorylated is consistent with another report that this protein is a potential target of PP1 using a differential proteome approach to identify hyperphosphorylated proteins in the PP1cc null mouse [19]. Many of the proteins identified in that study were also identified by our laboratory as part of the TLRR complex in testis including tubulin, actin, and proteosome subunits [6]. These results support the hypothesis that the complex containing TLRR and PP1 regulates the reversible phosphorylation of proteins important for spermiogenesis including cytoskeletal polymers, molecular motors, and chaperones. For example, phosphorylation of the motor domain of kinesin-1 by JNK3 has been shown to negatively regulate kinesin binding to microtubules therefore inhibiting kinesin-based transport [20].

In elongated spermatids, TLRR is localized near the centrosome, and thereafter is lost from spermatozoa as part of the residual body. The relocalization of this protein from the spermatid microtubule cytoskeleton in early spermatids to the centrosome in later stage spermatids is suggestive of a role in biogenesis of the sperm flagella. Supporting this hypothesis is the finding that TLRR (lrrc67) is expressed in other ciliated tissues [21] and has been identified as a member of the ciliome by comparative genomics [22]. Examination of the GeneAtlas GNF1M gcrma database for lrrc67 confirmed that this gene is expressed in ciliated tissues including olfactory epithelium and retina although it is expressed at a much higher level in testis (30-fold mean expression level compared to olfactory epithelium) [23]. Other investigators have reported that proper regulation of PP1 activity is necessary for stabilization of tubulin in primary cilia of retinal epithelial cells providing a link between PP1 and cilia [24].

Our report that TLRR interacts with PP1 in spermatocytes and round spermatids, before its translocation to the centrosome may indicate a bifunctional role for TLRR: early in spermiogenesis in conjunction with PP1 and later in this process independent of PP1. Further studies are underway to dissect the interaction of TLRR and PP1 isoforms *in vivo* in the various germ cells in the seminiferous epithelium.

## Materials and Methods

### Immunoprecipitation and co-immunoprecipitation

All use of animals was approved by the East Carolina University Brody School of Medicine Institutional Animal Care and Use Committee (protocol #W179c) in accordance with the *Guide for the Care and Use of Agricultural Animals in Agricultural Research and Teaching*. Mouse testes extract was prepared as previously described [9]. Briefly, decapsulated testes from adult or postnatal mice were homogenized in buffer B (10 mM Tris-HCl, pH 7.0, 1 mM EDTA, 1 mM EGTA, 10 mM benzamidine-HCl, 150 mM PMSF, and 0.1% (v/v) β-mercaptoethanol), supplemented with mammalian protease inhibitor cocktail (Sigma-Aldrich; St. Louis, MO), and centrifuged at 16,000×g to remove cellular debris. When lysate was to be used for determination of protein phosphorylation, Buffer B was also supplemented with phosphatase inhibitor cocktail set III (EMD Chemical, Inc.; Gibbstown, NJ). Protein concentration in tissue lysates was determined by the Coomassie brilliant blue method (Biorad; Hercules, CA). For co-immunoprecipitation experiments, the Trueblot kit from eBioscience (San Diego, CA) was used. Briefly, precleared testis lysate was incubated with anti-rabbit beads and antibody to either PP1-FL18 or PP1γ2. Beads were collected, washed, resuspended in Trueblot SDS buffer, and bound proteins separated by SDS-PAGE. Trueblot secondary antibody linked to HRP was used for detection according to manufacturer's instructions. For phosphatase assays, testis lysate (1.5–2 mg protein) was incubated with antibody-linked Sepharose beads overnight at 4°C. Immune complexes were collected by centrifugation, unbound protein removed, and the pellet washed extensively to remove nonspecifically bound protein. Bound proteins were eluted from the pellet with triethanolamine buffer (TEA; 50 mM triethanolamine, pH 11, 0.15 M sodium chloride, 0.1% Triton X-100) and immediately diluted in phosphatase reaction buffer (Invitrogen; Carlsbad, CA) for further analysis (see below). Negative control for co-immunoprecipitation with TLRR or PP1 antibodies was normal rabbit IgG (NRIgG) bound to beads. For experiments to determine whether TLRR, kinesin-1B, or tubulin are phosphorylated, testis extract was incubated with Sepharose beads linked to 12 µg anti-phosphoserine antibodies (mixture of clones 1CB, 4A3, 4A9, and 16B3 from EMD Chemical, Inc.; Gibbstown, NJ) or control beads linked to an equivalent amount of normal mouse IgG (NMIgG).

### Phosphatase assays

Samples were prepared by immunoprecipitation with the TLRR antibody as described above or with NRIgG for negative control. Phosphatase activity was measured in each immunoprecipitate using either the EnzChek® Phosphatase Assay kit for total phosphatase activity (Invitrogen; Carlsbad, CA) or the RediPlate 96 EnzChek® Serine/Threonine Phosphatase Assay kit (Invitrogen; Carlsbad, CA) under conditions optimal for PP1. The fluorescent product of the reaction was measured after a 60 minute incubation at room temperature using a microplate reader (360 nm excitation, 460 nm emission). Negative control for phosphatase activity was buffer substituted for the experimental sample and positive control was potato acid phosphatase. In order to calculate the relative fluorescence at each developmental time point, the amount of fluorescence in the control immunoprecipitate was subtracted from the TLRR immunoprecipitate and the results displayed as percentage of the activity at 56 days. Statistical significance was assessed using a Tukey's test.

### Western blot

Protein samples from immunoprecipitation experiments or protein purification fractions were separated by polyacrylamide gel electrophoresis (PAGE) through 10% acrylamide gels or precast 4–20% acrylamide gels (Thermo Scientific; Rockford IL), equilibrated in and electrophoretically transferred from the gel matrix to PVDF membrane (BioRad Laboratories; Hercules CA) in Towbin transfer buffer. Proteins were detected on the membrane with affinity purified TLRR antibody prepared as previously described at a dilution of 1∶5,000 [5]. Other antibodies used for western blot in these experiments were the pan-PP1 antibodies PP1-FL18 and PP1-E9 (both at 1∶250; Santa Cruz Biotechnology, Inc, Santa Cruz, CA), anti-PP1γ2 (1∶5,000; kind gift of Dr. S. Vijayaraghavan, Kent State University) [25], UIC 81 specific for kinesin-1B (1∶250, kind gift of Dr. S. Brady, University of Illinois, Chicago) [26], and anti-actin (20–33) (1∶200; Sigma Aldrich, St. Louis, MO). Immune complexes bound to the membrane were detected with horseradish peroxidase-conjugated donkey secondary antibody (Jackson ImmunoResearch Inc.; West Grove, PA) diluted 1∶40,000 in TTBS (100 mM Tris, pH 7.5, 150 mM NaCl, 0.1% Tween 20) and developed with enhanced chemiluminescent reagents as described by the manufacturer (Thermo Scientific; Rockford IL).

### DNA manipulations

Plasmids containing coding sequence for mouse PP1γ1 and PP1γ2 were generous gifts of Dr. S. Vijayaraghavan, Kent State University. Human PP1α cloned into pGBKT7 was a kind gift of Dr. Susannah Varmuza, University of Toronto. All three PP1 genes and TLRR were transferred to Gateway vector (Invitrogen; Carlsbad CA) pDEST17 for bacterial expression of His6 fusion proteins. PP1α was amplified with CACCCCGACAGCGAGAAGCTCAAC as the 5′ primer and CTATTTCTTGGCTTTGGCAGAATT as the 3′ primer. PP1γ1 was amplified with CACCCGGATATCGACAAACTCAACATCGAC as the 5′ primer and CTATTTCTTTGCTTGCTTTGTGATC as the 3′ primer while PP1γ2 was amplified with the same 5′ primer as for PP1γ1 and TCACTCGTATAGGACAGTGTTG as the 3′ primer. TLRR was amplified with CACCTTCGACTGACGGTGGATTTAATTG as the 5′ primer and GGAAAATTTGTCTGAGAAAAAGGAGTAA as the 3′ primer.

### Purification of His6 fusion proteins

Expression constructs were transformed into BL21-AI strain (Invitrogen; Carlsbad, CA) where T7 polymerase is under control of the *araBAD* promoter. Cultures, typically 500 ml, were grown until mid-log (OD600 = 0.4–0.6) and arabinose added to a final concentration of 0.2% to induce expression of the recombinant protein. All growth media was supplemented with 0.1% glucose to fully repress basal levels of expression. After 2–4 hours of growth, cells were collected by centrifugation and the cell pellet frozen overnight at −80°C. The cell pellet was then resuspended in lysis buffer (50 mM potassium phosphate, pH 7.8, 400 mM NaCl, 100 mM KCl, 10% glycerol, 0.5% Triton X-100, 10 mM imidazole, 5 mM β-mercaptoethanol) containing 1 mg/ml lysozyme, lysed by sonication, and the cell debris removed by centrifugation. The cleared lysate was mixed with Ni-NTA agarose (Qiagen; Valencia, CA) for 1 hour at 4°C, the slurry transferred to a disposable column, and the flow-through collected. The resin was washed twice with 10 column volumes of wash buffer (50 mM sodium phosphate, 300 mM sodium chloride, 20 mM imidazole, 10% glycerol, 0.5% Triton X-100, 5 mM β-mercaptoethanol) and His6 tagged proteins eluted with elution buffer (50 mM sodium phosphate, 300 mM sodium chloride, 250 mM imidazole, 10% glycerol, 0.5% Triton-X100, 5 mM β-mercaptoethanol). All purification buffers were supplemented with 150 mM PMSF and bacterial protease inhibitor cocktail (Sigma-Aldrich; St. Louis, MO). Protein concentration was determined using the Coomassie brilliant blue method (Biorad; Hercules, CA), protein verified by western blot with appropriate antibodies and stored in aliquots in protein stabilization cocktail (Thermo Scientific; Rockford, IL).
